# {Bis[2-(3,5-dimethyl­pyrazol-1-yl-κ*N* ^2^)eth­yl]amine-κ*N*}chloridopalladium(II) chloride 0.25-hydrate

**DOI:** 10.1107/S1600536810035427

**Published:** 2010-09-11

**Authors:** Ilia A. Guzei, Lara C. Spencer, Nangamso Miti, James Darkwa

**Affiliations:** a Department of Chemistry, University of Wisconsin-Madison, 1101 University Ave, Madison, WI 53706, USA; b Department of Chemistry, University of Johannesburg, Auckland Park Kingsway Campus, Auckland Park 2006, South Africa

## Abstract

The title compound, [PdCl(C_14_H_23_N_5_)]Cl·0.25H_2_O, is a pseudopolymorph of the previously reported compound [PdCl(C_14_H_23_N_5_)]Cl·2H_2_O [de Mendoza *et al.* (2006 ▶). *Acta Cryst.* E**62**, m2934–m2936]. The cationic complex and chloride anion are disordered over two positions each in a 0.584 (4):0.416 (4) ratio. The geometry about the Pd atom is distorted square-planar. The pyrazole rings are almost perpendicular, forming a dihedral angle of 86.6 (6)° to each other, to mitigate steric conflict between their methyl groups.

## Related literature

For the previously reported pseudopolymorph, see: de Mendoza *et al.* (2006 ▶). For the use of bis­(pyrazol­yl)alkyl­amines as ligands in metal complexes, see: Kunrath *et al.* (2003 ▶); Ajellal *et al.* (2006 ▶); Zhang *et al.* (2008 ▶); John *et al.* (2010 ▶). For geometrical parameter checks, see: Bruno *et al.* (2004 ▶); Allen (2002 ▶).
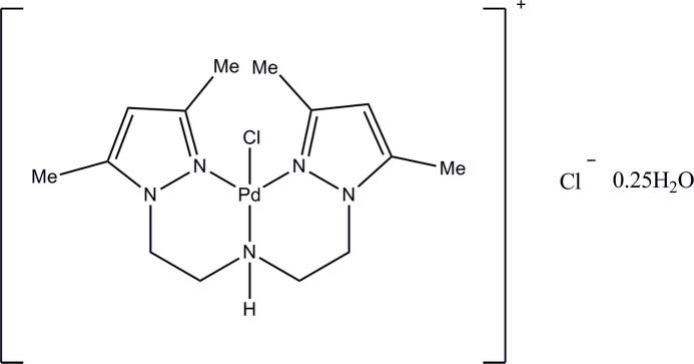


## Experimental

### 

#### Crystal data

[PdCl(C_14_H_23_N_5_)]Cl·0.25H_2_O
*M* *_r_* = 443.68
Monoclinic, 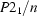

*a* = 10.5995 (8) Å
*b* = 12.4740 (9) Å
*c* = 13.8168 (10) Å
β = 99.865 (1)°
*V* = 1799.8 (2) Å^3^
*Z* = 4
Mo *K*α radiation
μ = 1.33 mm^−1^
*T* = 100 K
0.27 × 0.27 × 0.19 mm

#### Data collection

Bruker CCD 1000 area-detector diffractometer
Absorption correction: multi-scan (*SADABS*; Bruker, 2000 ▶) *T*_min_ = 0.715, *T*_max_ = 0.786
26455 measured reflections
5182 independent reflections
4450 reflections with *I* > 2σ(*I*)
*R*_int_ = 0.029

#### Refinement

*R*[*F* ^2^ > 2σ(*F* ^2^)] = 0.023
*wR*(*F* ^2^) = 0.057
*S* = 1.01
5182 reflections
415 parameters
691 restraints
H-atom parameters constrained
Δρ_max_ = 0.44 e Å^−3^
Δρ_min_ = −0.28 e Å^−3^

### 

Data collection: *SMART* (Bruker, 2000 ▶); cell refinement: *SAINT-Plus* (Bruker, 2007 ▶); data reduction: *SAINT-Plus*; program(s) used to solve structure: *SHELXS97* (Sheldrick, 2008 ▶); program(s) used to refine structure: *SHELXL97* (Sheldrick, 2008 ▶) and *FCF*_filter (Guzei, 2007 ▶); molecular graphics: *SHELXTL* (Sheldrick, 2008 ▶) and *DIAMOND* (Brandenburg, 2009 ▶); software used to prepare material for publication: *SHELXTL*, *publCIF* (Westrip, 2010 ▶) and *modiCIFer* (Guzei, 2007 ▶).

## Supplementary Material

Crystal structure: contains datablocks global, I. DOI: 10.1107/S1600536810035427/su2207sup1.cif

Structure factors: contains datablocks I. DOI: 10.1107/S1600536810035427/su2207Isup2.hkl

Additional supplementary materials:  crystallographic information; 3D view; checkCIF report

## Figures and Tables

**Table 1:** Hydrogen-bond geometry (Å, °)

*D*—H⋯*A*	*D*—H	H⋯*A*	*D*⋯*A*	*D*—H⋯*A*
N3—H3⋯Cl2	0.93	2.24	3.162 (12)	174
N3*A*—H3*B*⋯Cl2*A*	0.93	2.17	3.088 (15)	170

**Fig. 1.**
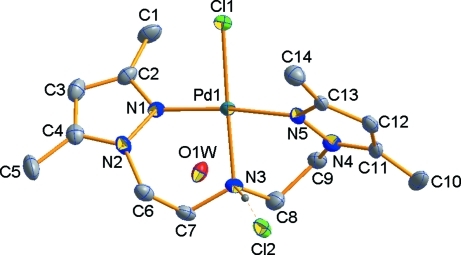
Molecular structure of the title compond. The thermal ellipsoids are shown at the 50% probability level. All hydrogen atoms connected to carbon atoms and the minor components of disordered atoms were omitted for clarity. The intermolecular hydrogen bond is shown as a dashed line. The solvent water molecule has an ocupancy of 25%.

## supplementary crystallographic information

### Comment

Bis(pyrazolyl)alkylamines are frequently used as N^N^N ligands in metal complexes (Kunrath *et al.*, 2003, Ajellal *et al.*, 2006, Zhang *et al.*, 2008), although bis(pyrazolyl)arylamines can act as bidentate N^N ligands (John *et al.*, 2010).

Palladium dichloride reacts with bis(3,5-dimethylpyrazolyl)alkylamine to afford the title compound, in which the ligand acts as a tridentate N^N^N donor. It is a pseudopolymorph of the previously reported complex [PdCl(C_14_H_23_N_5_]Cl.2H_2_O, which contains two solvent water molecules per ionic complex (de Mendoza *et al.*, 2006).

The title compound consists of discrete [PdCl(C_14_H_23_N_5_)] cations and chloride anions (Fig. 1). The lattice also contains one quarter of a solvent water molecule per ionic complex. The geometry about the central palladium atom is slightly distorted square planar. This distortion is observed in the bond angles about the palladium atom and the deviation from planarity by the palladium and its four coordinating atoms (average r.m.s. of 0.07 (6) Å). The pyrazole rings are almost perpendicular to each other forming an average dihedral angle of 86.6 (6)° required to mitigate steric conflict between the methyl groups C1 and C14. The pyrazole rings are tilted av. 47.2 (11)° relative to the Pd coordination plane. The bond distances and angles are typical, as confirmed by a *Mogul* structural check (Bruno *et al.*, 2004) and by comparing the values in the title compound to six similar compounds found in the Cambridge Structural Database (CSD, version 5.31, last update May 2010; Allen, 2002).

The cationic complex exhibits a "whole molecule disorder" over two positions, and the chloride anion is also disordered over two positions. The major components of the disordered moieties are present 58.4 (4)% of the time. There is one strong intermolecular hydrogen bond of the type N—H···Cl present in the ionic compound (Table 1). It is likely that when the solvent water molecule is present it participates in a hydrogen bond with atom Cl2A as indicated by the distance of 3.034 (5) Å between the two atoms. Since the hydrogen atoms on the solvent water molecule could not be located, no further information about a possible hydrogen bond can be obtained. The solvent water molecule cannot be present when atom Cl2, the major component of the disordered chloride anion, is present as it would place the O and Cl atoms in prohibitively close proximity.

### Experimental

A solution of [PdCl_2_(NCMe)_2_] (0.10 g, 0.39 mmol) and bis(3,5-dimethylpyrazolyl)ethylamine (0.10 g, 0.39 mmol) in dichloromethane (20 ml) was stirred at 233 K for 24 h. The resultant yellowish-orange solution was stored at 269 K for several days to form orange crystals. Yield: 0.06 g (35%). 1^H^ NMR (CDCl_3_): 2.49 (s, 12H, CH_3_, pz), 3.62 (t, ^3^*J_H—H_* = 12.3 Hz, 4H, CH_2_-pz), 4.00 (t, ^3^*J_H—H_* = 12.3 Hz, 4H, CH_2_—NH), 5.26 (s, 2H, CH, pz).

### Refinement

The cationic palladium complex and chloride anion are disordered over two positions in a 0.584 (4):0.416 (4) ratio. The complexes were refined with similarity restraints. There is also one quarter molecule of a solvent water per molecule of complex. The H-atoms of this solvent water could not be located. All the other H-atoms were placed in idealized locations and refined as riding: N-H = 0.93 Å, C-H = 0.95, 0.99 and 0.98 Å for CH, CH_2_ and CH_3_ H-atoms, respectively, with *U*_iso_(H) = k × *U*_eq_(bearing atom), where k = 1.5 methyl H-atoms and k = 1.2 for all other H–atoms.

### Crystal data

**Table:**

[PdCl(C_14_H_23_N_5_)]Cl·0.25H_2_O	*F*(000) = 898
*M**_r_* = 443.68	*D*_x_ = 1.636 Mg m^−^^3^
Monoclinic, *P*2_1_/*n*	Mo *K*α radiation, λ = 0.71073 Å
Hall symbol: -P 2yn	Cell parameters from 11031 reflections
*a* = 10.5995 (8) Å	θ = 2.2–30.0°
*b* = 12.4740 (9) Å	µ = 1.33 mm^−^^1^
*c* = 13.8168 (10) Å	*T* = 100 K
β = 99.865 (1)°	Block, yellow
*V* = 1799.8 (2) Å^3^	0.27 × 0.27 × 0.19 mm
*Z* = 4	

### Data collection

**Table:**

Bruker CCD 1000 area-detector diffractometer	5182 independent reflections
Radiation source: fine-focus sealed tube	4450 reflections with *I* > 2σ(*I*)
graphite	*R*_int_ = 0.029
0.30° ω and 0.4 ° φ scans	θ_max_ = 30.0°, θ_min_ = 2.2°
Absorption correction: multi-scan (*SADABS*; Bruker, 2000)	*h* = −14→14
*T*_min_ = 0.715, *T*_max_ = 0.786	*k* = −17→17
26455 measured reflections	*l* = −18→19

### Refinement

**Table:**

Refinement on *F*^2^	Primary atom site location: structure-invariant direct methods
Least-squares matrix: full	Secondary atom site location: difference Fourier map
*R*[*F*^2^ > 2σ(*F*^2^)] = 0.023	Hydrogen site location: inferred from neighbouring sites
*wR*(*F*^2^) = 0.057	H-atom parameters constrained
*S* = 1.01	*w* = 1/[σ^2^(*F*_o_^2^) + (0.0271*P*)^2^ + 0.7513*P*] where *P* = (*F*_o_^2^ + 2*F*_c_^2^)/3
5182 reflections	(Δ/σ)_max_ = 0.001
415 parameters	Δρ_max_ = 0.44 e Å^−^^3^
691 restraints	Δρ_min_ = −0.28 e Å^−^^3^

### Special details

**Table:**

Geometry. All e.s.d.'s (except the e.s.d. in the dihedral angle between two l.s. planes) are estimated using the full covariance matrix. The cell e.s.d.'s are taken into account individually in the estimation of e.s.d.'s in distances, angles and torsion angles; correlations between e.s.d.'s in cell parameters are only used when they are defined by crystal symmetry. An approximate (isotropic) treatment of cell e.s.d.'s is used for estimating e.s.d.'s involving l.s. planes.
Refinement. Refinement of *F*^2^ against ALL reflections. The weighted *R*-factor *wR* and goodness of fit *S* are based on *F*^2^, conventional *R*-factors *R* are based on *F*, with *F* set to zero for negative *F*^2^. The threshold expression of *F*^2^ > σ(*F*^2^) is used only for calculating *R*-factors(gt) *etc*. and is not relevant to the choice of reflections for refinement. *R*-factors based on *F*^2^ are statistically about twice as large as those based on *F*, and *R*- factors based on ALL data will be even larger.

### Fractional atomic coordinates and isotropic or equivalent isotropic displacement parameters (Å^2^)

**Table:**

	*x*	*y*	*z*	*U*_iso_*/*U*_eq_	Occ. (<1)
Pd1	0.80405 (12)	0.79001 (8)	1.01257 (6)	0.01812 (12)	0.584 (4)
Cl1	0.72843 (17)	0.72421 (12)	1.14792 (8)	0.0288 (3)	0.584 (4)
Cl2	0.67632 (16)	0.72034 (5)	0.71803 (7)	0.0260 (3)	0.584 (4)
N1	0.7742 (3)	0.9419 (3)	1.0504 (3)	0.0226 (6)	0.584 (4)
N2	0.7043 (4)	1.0067 (3)	0.9815 (3)	0.0227 (7)	0.584 (4)
N3	0.8628 (7)	0.8406 (9)	0.8864 (7)	0.0196 (12)	0.584 (4)
H3	0.8127	0.8008	0.8374	0.024*	0.584 (4)
N4	0.9397 (10)	0.6260 (7)	0.9251 (9)	0.0213 (12)	0.584 (4)
N5	0.8247 (14)	0.6423 (8)	0.9573 (12)	0.0211 (18)	0.584 (4)
C1	0.8615 (4)	0.9538 (4)	1.2288 (3)	0.0343 (8)	0.584 (4)
H1A	0.8131	0.8997	1.2586	0.051*	0.584 (4)
H1B	0.8834	1.0134	1.2748	0.051*	0.584 (4)
H1C	0.9402	0.9217	1.2137	0.051*	0.584 (4)
C2	0.7825 (4)	0.9942 (4)	1.1371 (3)	0.0245 (8)	0.584 (4)
C3	0.7174 (5)	1.0910 (5)	1.1218 (4)	0.0304 (9)	0.584 (4)
H3A	0.7074	1.1427	1.1704	0.036*	0.584 (4)
C4	0.6704 (5)	1.0979 (4)	1.0231 (4)	0.0266 (8)	0.584 (4)
C5	0.5964 (6)	1.1848 (5)	0.9651 (5)	0.0409 (11)	0.584 (4)
H5A	0.6469	1.2144	0.9182	0.061*	0.584 (4)
H5B	0.5777	1.2416	1.0095	0.061*	0.584 (4)
H5C	0.5160	1.1556	0.9292	0.061*	0.584 (4)
C6	0.7008 (4)	0.9829 (4)	0.8770 (4)	0.0238 (9)	0.584 (4)
H6A	0.6694	1.0465	0.8372	0.029*	0.584 (4)
H6B	0.6405	0.9231	0.8570	0.029*	0.584 (4)
C7	0.8313 (5)	0.9530 (5)	0.8579 (4)	0.0235 (9)	0.584 (4)
H7A	0.8344	0.9625	0.7872	0.028*	0.584 (4)
H7B	0.8959	1.0013	0.8956	0.028*	0.584 (4)
C8	0.9946 (5)	0.8097 (5)	0.8785 (4)	0.0286 (10)	0.584 (4)
H8A	1.0532	0.8700	0.9008	0.034*	0.584 (4)
H8B	0.9996	0.7950	0.8088	0.034*	0.584 (4)
C9	1.0378 (8)	0.7104 (5)	0.9401 (6)	0.0259 (12)	0.584 (4)
H9A	1.1180	0.6826	0.9218	0.031*	0.584 (4)
H9B	1.0558	0.7304	1.0104	0.031*	0.584 (4)
C10	1.0477 (4)	0.4927 (4)	0.8342 (2)	0.0369 (8)	0.584 (4)
H10A	1.0551	0.5399	0.7787	0.055*	0.584 (4)
H10B	1.0302	0.4193	0.8102	0.055*	0.584 (4)
H10C	1.1281	0.4940	0.8813	0.055*	0.584 (4)
C11	0.9421 (5)	0.5300 (4)	0.8827 (4)	0.0218 (9)	0.584 (4)
C12	0.8283 (4)	0.4817 (4)	0.8913 (3)	0.0233 (7)	0.584 (4)
H12	0.8009	0.4123	0.8685	0.028*	0.584 (4)
C13	0.7602 (7)	0.5541 (6)	0.9401 (5)	0.0233 (11)	0.584 (4)
C14	0.6270 (4)	0.5440 (4)	0.9603 (3)	0.0325 (8)	0.584 (4)
H14A	0.6253	0.5644	1.0286	0.049*	0.584 (4)
H14B	0.5982	0.4696	0.9498	0.049*	0.584 (4)
H14C	0.5701	0.5912	0.9160	0.049*	0.584 (4)
Pd1A	0.77404 (15)	0.77658 (11)	1.00983 (9)	0.01597 (15)	0.416 (4)
Cl1A	0.68638 (18)	0.69209 (17)	1.13068 (12)	0.0258 (3)	0.416 (4)
Cl2A	0.7350 (3)	0.72156 (8)	0.69987 (10)	0.0311 (5)	0.416 (4)
N1A	0.7336 (4)	0.9221 (4)	1.0594 (4)	0.0195 (8)	0.416 (4)
N2A	0.6665 (5)	0.9933 (5)	0.9944 (4)	0.0219 (9)	0.416 (4)
N3A	0.8351 (11)	0.8443 (12)	0.8917 (9)	0.0164 (12)	0.416 (4)
H3B	0.7974	0.8038	0.8379	0.020*	0.416 (4)
N4A	0.9562 (12)	0.6458 (9)	0.9264 (12)	0.0152 (14)	0.416 (4)
N5A	0.8447 (19)	0.6399 (11)	0.9623 (16)	0.0165 (17)	0.416 (4)
C1A	0.8271 (5)	0.9143 (4)	1.2373 (4)	0.0289 (10)	0.416 (4)
H1D	0.7743	0.8595	1.2619	0.043*	0.416 (4)
H1E	0.8529	0.9682	1.2885	0.043*	0.416 (4)
H1F	0.9036	0.8806	1.2197	0.043*	0.416 (4)
C2A	0.7510 (5)	0.9674 (5)	1.1478 (4)	0.0212 (10)	0.416 (4)
C3A	0.6937 (7)	1.0679 (6)	1.1386 (6)	0.0252 (11)	0.416 (4)
H3C	0.6909	1.1175	1.1904	0.030*	0.416 (4)
C4A	0.6421 (6)	1.0829 (5)	1.0422 (5)	0.0238 (11)	0.416 (4)
C5A	0.5703 (9)	1.1756 (7)	0.9907 (6)	0.0428 (16)	0.416 (4)
H5D	0.6213	1.2085	0.9457	0.064*	0.416 (4)
H5E	0.5539	1.2288	1.0392	0.064*	0.416 (4)
H5F	0.4887	1.1504	0.9533	0.064*	0.416 (4)
C6A	0.6608 (6)	0.9767 (6)	0.8892 (5)	0.0198 (11)	0.416 (4)
H6C	0.6234	1.0407	0.8527	0.024*	0.416 (4)
H6D	0.6053	0.9144	0.8672	0.024*	0.416 (4)
C7A	0.7925 (6)	0.9571 (6)	0.8680 (6)	0.0190 (12)	0.416 (4)
H7C	0.8533	1.0073	0.9072	0.023*	0.416 (4)
H7D	0.7936	0.9714	0.7976	0.023*	0.416 (4)
C8A	0.9775 (8)	0.8360 (6)	0.8929 (6)	0.0229 (12)	0.416 (4)
H8C	1.0202	0.9013	0.9233	0.027*	0.416 (4)
H8D	0.9939	0.8315	0.8246	0.027*	0.416 (4)
C9A	1.0336 (11)	0.7383 (7)	0.9498 (9)	0.0220 (15)	0.416 (4)
H9C	1.1202	0.7243	0.9348	0.026*	0.416 (4)
H9D	1.0424	0.7530	1.0211	0.026*	0.416 (4)
C10A	1.0903 (4)	0.5384 (4)	0.8307 (3)	0.0243 (8)	0.416 (4)
H10D	1.0891	0.5889	0.7762	0.036*	0.416 (4)
H10E	1.0908	0.4648	0.8059	0.036*	0.416 (4)
H10F	1.1673	0.5505	0.8799	0.036*	0.416 (4)
C11A	0.9752 (7)	0.5549 (6)	0.8762 (5)	0.0184 (11)	0.416 (4)
C12A	0.8706 (6)	0.4902 (5)	0.8755 (4)	0.0197 (10)	0.416 (4)
H12A	0.8595	0.4204	0.8478	0.024*	0.416 (4)
C13A	0.7836 (8)	0.5442 (8)	0.9224 (7)	0.0197 (14)	0.416 (4)
C14A	0.6521 (5)	0.5147 (5)	0.9364 (4)	0.0298 (11)	0.416 (4)
H14D	0.6495	0.5092	1.0068	0.045*	0.416 (4)
H14E	0.6285	0.4456	0.9047	0.045*	0.416 (4)
H14F	0.5917	0.5699	0.9070	0.045*	0.416 (4)
O1W	0.5114 (4)	0.7644 (3)	0.8052 (3)	0.0239 (8)	0.25

### Atomic displacement parameters (Å^2^)

**Table:**

	*U*^11^	*U*^22^	*U*^33^	*U*^12^	*U*^13^	*U*^23^
Pd1	0.0221 (3)	0.0185 (2)	0.01357 (11)	0.00310 (19)	0.0024 (2)	0.00027 (12)
Cl1	0.0386 (6)	0.0284 (5)	0.0217 (4)	0.0100 (4)	0.0115 (4)	0.0077 (3)
Cl2	0.0367 (6)	0.0176 (3)	0.0212 (3)	−0.0021 (3)	−0.0023 (3)	−0.0008 (2)
N1	0.0281 (18)	0.0243 (14)	0.0150 (11)	0.0044 (12)	0.0020 (12)	−0.0012 (9)
N2	0.025 (2)	0.0190 (14)	0.0216 (15)	0.0028 (13)	−0.0026 (12)	0.0003 (10)
N3	0.019 (3)	0.0227 (17)	0.0169 (13)	−0.001 (2)	0.0025 (16)	0.0033 (11)
N4	0.021 (2)	0.022 (3)	0.0189 (14)	−0.0030 (17)	−0.0023 (15)	−0.002 (2)
N5	0.018 (4)	0.0246 (19)	0.020 (2)	0.0068 (17)	0.002 (3)	0.0044 (14)
C1	0.0361 (19)	0.048 (3)	0.0175 (13)	−0.0009 (15)	0.0020 (12)	−0.0069 (15)
C2	0.0242 (19)	0.029 (2)	0.0202 (15)	−0.0046 (13)	0.0044 (12)	−0.0081 (13)
C3	0.031 (2)	0.023 (2)	0.039 (2)	−0.0032 (15)	0.0121 (15)	−0.0087 (16)
C4	0.022 (2)	0.0230 (18)	0.037 (2)	−0.0017 (13)	0.0088 (14)	0.0004 (14)
C5	0.040 (2)	0.0256 (18)	0.061 (3)	0.0132 (15)	0.0178 (19)	0.009 (2)
C6	0.028 (3)	0.0209 (14)	0.0195 (16)	−0.0031 (18)	−0.0034 (16)	0.0009 (11)
C7	0.032 (3)	0.0215 (15)	0.0156 (16)	−0.0040 (19)	−0.0001 (16)	0.0030 (11)
C8	0.0254 (19)	0.033 (3)	0.028 (2)	0.0020 (18)	0.0088 (13)	0.0016 (17)
C9	0.0310 (17)	0.027 (3)	0.0191 (19)	0.002 (2)	0.0020 (13)	0.002 (2)
C10	0.0388 (19)	0.053 (2)	0.0182 (12)	0.0216 (16)	0.0019 (12)	−0.0002 (14)
C11	0.025 (3)	0.022 (3)	0.0175 (14)	0.0099 (17)	0.0012 (16)	−0.0002 (16)
C12	0.031 (2)	0.0193 (15)	0.0172 (16)	0.0045 (16)	−0.0035 (13)	0.0013 (12)
C13	0.034 (3)	0.0192 (16)	0.017 (2)	0.0105 (16)	0.0048 (14)	0.0032 (14)
C14	0.0299 (18)	0.033 (2)	0.034 (2)	−0.0002 (14)	0.0056 (13)	0.0060 (14)
Pd1A	0.0168 (4)	0.0158 (3)	0.01479 (16)	0.0016 (2)	0.0015 (2)	0.00192 (15)
Cl1A	0.0246 (6)	0.0282 (7)	0.0266 (5)	0.0077 (5)	0.0097 (5)	0.0115 (5)
Cl2A	0.0471 (11)	0.0213 (5)	0.0215 (5)	0.0048 (5)	−0.0038 (5)	−0.0035 (3)
N1A	0.016 (2)	0.025 (2)	0.0169 (15)	0.0068 (14)	0.0022 (14)	0.0038 (13)
N2A	0.020 (2)	0.023 (2)	0.0231 (19)	0.0004 (17)	0.0029 (16)	0.0017 (14)
N3A	0.014 (4)	0.0178 (19)	0.016 (2)	0.000 (2)	−0.0004 (19)	−0.0007 (15)
N4A	0.016 (3)	0.015 (3)	0.0155 (19)	0.001 (2)	0.005 (2)	−0.004 (2)
N5A	0.012 (4)	0.015 (2)	0.021 (3)	0.0065 (18)	−0.003 (3)	0.0012 (17)
C1A	0.035 (3)	0.033 (3)	0.0186 (17)	0.0070 (18)	0.0032 (17)	−0.0008 (18)
C2A	0.021 (2)	0.023 (3)	0.0217 (18)	0.0015 (17)	0.0075 (16)	0.0012 (16)
C3A	0.028 (3)	0.022 (3)	0.028 (2)	−0.0028 (19)	0.0124 (18)	−0.0037 (18)
C4A	0.022 (3)	0.016 (2)	0.037 (3)	0.0018 (18)	0.0167 (18)	0.0000 (18)
C5A	0.056 (4)	0.031 (3)	0.048 (4)	0.021 (3)	0.028 (3)	0.013 (2)
C6A	0.018 (3)	0.019 (2)	0.021 (2)	0.0039 (19)	0.0006 (18)	0.0056 (14)
C7A	0.024 (4)	0.0173 (19)	0.015 (2)	−0.003 (2)	0.002 (2)	0.0025 (14)
C8A	0.032 (3)	0.017 (3)	0.020 (2)	0.0082 (19)	0.0079 (18)	0.0021 (18)
C9A	0.022 (2)	0.016 (3)	0.026 (3)	−0.002 (2)	−0.0006 (17)	−0.004 (2)
C10A	0.025 (2)	0.027 (2)	0.0230 (17)	0.0089 (15)	0.0092 (14)	0.0037 (15)
C11A	0.024 (3)	0.017 (3)	0.0131 (17)	−0.0023 (17)	−0.0006 (18)	0.0052 (16)
C12A	0.026 (3)	0.017 (2)	0.0171 (19)	0.003 (2)	0.0065 (19)	0.0016 (14)
C13A	0.019 (3)	0.024 (3)	0.016 (3)	−0.001 (2)	0.0010 (19)	0.001 (2)
C14A	0.025 (2)	0.036 (3)	0.028 (3)	−0.0013 (19)	0.0044 (17)	0.0051 (19)
O1W	0.0207 (18)	0.024 (2)	0.028 (2)	−0.0029 (14)	0.0055 (15)	−0.0101 (15)

### Geometric parameters (Å, °)

**Table:**

Pd1—N1	2.005 (4)	Pd1A—N1A	2.013 (5)
Pd1—N5	2.021 (8)	Pd1A—N5A	2.017 (10)
Pd1—N3	2.048 (6)	Pd1A—N3A	2.039 (8)
Pd1—Cl1	2.3079 (12)	Pd1A—Cl1A	2.3009 (16)
N1—C2	1.353 (5)	N1A—C2A	1.328 (8)
N1—N2	1.367 (6)	N1A—N2A	1.372 (8)
N2—C4	1.350 (7)	N2A—C4A	1.345 (10)
N2—C6	1.468 (6)	N2A—C6A	1.460 (9)
N3—C8	1.471 (7)	N3A—C7A	1.497 (14)
N3—C7	1.479 (11)	N3A—C8A	1.511 (11)
N3—H3	0.9300	N3A—H3B	0.9300
N4—C11	1.335 (9)	N4A—N5A	1.360 (19)
N4—N5	1.381 (14)	N4A—C11A	1.362 (13)
N4—C9	1.470 (9)	N4A—C9A	1.421 (12)
N5—C13	1.296 (14)	N5A—C13A	1.423 (17)
C1—C2	1.483 (6)	C1A—C2A	1.510 (8)
C1—H1A	0.9800	C1A—H1D	0.9800
C1—H1B	0.9800	C1A—H1E	0.9800
C1—H1C	0.9800	C1A—H1F	0.9800
C2—C3	1.388 (8)	C2A—C3A	1.390 (10)
C3—C4	1.372 (8)	C3A—C4A	1.364 (11)
C3—H3A	0.9500	C3A—H3C	0.9500
C4—C5	1.489 (8)	C4A—C5A	1.496 (11)
C5—H5A	0.9800	C5A—H5D	0.9800
C5—H5B	0.9800	C5A—H5E	0.9800
C5—H5C	0.9800	C5A—H5F	0.9800
C6—C7	1.500 (5)	C6A—C7A	1.495 (7)
C6—H6A	0.9900	C6A—H6C	0.9900
C6—H6B	0.9900	C6A—H6D	0.9900
C7—H7A	0.9900	C7A—H7C	0.9900
C7—H7B	0.9900	C7A—H7D	0.9900
C8—C9	1.528 (9)	C8A—C9A	1.515 (12)
C8—H8A	0.9900	C8A—H8C	0.9900
C8—H8B	0.9900	C8A—H8D	0.9900
C9—H9A	0.9900	C9A—H9C	0.9900
C9—H9B	0.9900	C9A—H9D	0.9900
C10—C11	1.476 (6)	C10A—C11A	1.479 (8)
C10—H10A	0.9800	C10A—H10D	0.9800
C10—H10B	0.9800	C10A—H10E	0.9800
C10—H10C	0.9800	C10A—H10F	0.9800
C11—C12	1.372 (5)	C11A—C12A	1.371 (6)
C12—C13	1.399 (5)	C12A—C13A	1.389 (7)
C12—H12	0.9500	C12A—H12A	0.9500
C13—C14	1.490 (7)	C13A—C14A	1.486 (9)
C14—H14A	0.9800	C14A—H14D	0.9800
C14—H14B	0.9800	C14A—H14E	0.9800
C14—H14C	0.9800	C14A—H14F	0.9800
			
N1—Pd1—N5	172.9 (5)	C7A—N3A—C8A	109.2 (9)
N1—Pd1—N3	90.9 (3)	C7A—N3A—Pd1A	116.1 (6)
N5—Pd1—N3	83.7 (5)	C8A—N3A—Pd1A	114.6 (7)
N1—Pd1—Cl1	91.86 (10)	C7A—N3A—H3B	105.2
N5—Pd1—Cl1	93.3 (4)	C8A—N3A—H3B	105.2
N3—Pd1—Cl1	175.9 (4)	Pd1A—N3A—H3B	105.2
C2—N1—N2	106.1 (4)	N5A—N4A—C11A	110.8 (8)
C2—N1—Pd1	134.2 (3)	N5A—N4A—C9A	117.7 (11)
N2—N1—Pd1	117.9 (3)	C11A—N4A—C9A	131.4 (9)
C4—N2—N1	110.7 (4)	N4A—N5A—C13A	105.7 (9)
C4—N2—C6	128.7 (4)	N4A—N5A—Pd1A	117.8 (11)
N1—N2—C6	119.0 (4)	C13A—N5A—Pd1A	131.7 (12)
C8—N3—C7	113.7 (6)	C2A—C1A—H1D	109.5
C8—N3—Pd1	114.5 (5)	C2A—C1A—H1E	109.5
C7—N3—Pd1	115.5 (5)	H1D—C1A—H1E	109.5
C8—N3—H3	103.7	C2A—C1A—H1F	109.5
C7—N3—H3	103.7	H1D—C1A—H1F	109.5
Pd1—N3—H3	103.7	H1E—C1A—H1F	109.5
C11—N4—N5	110.9 (7)	N1A—C2A—C3A	108.1 (6)
C11—N4—C9	129.6 (8)	N1A—C2A—C1A	122.5 (5)
N5—N4—C9	119.5 (8)	C3A—C2A—C1A	129.4 (6)
C13—N5—N4	106.5 (6)	C4A—C3A—C2A	107.9 (7)
C13—N5—Pd1	139.0 (8)	C4A—C3A—H3C	126.0
N4—N5—Pd1	114.5 (8)	C2A—C3A—H3C	126.0
N1—C2—C3	109.2 (4)	N2A—C4A—C3A	106.7 (7)
N1—C2—C1	122.3 (4)	N2A—C4A—C5A	122.3 (7)
C3—C2—C1	128.2 (4)	C3A—C4A—C5A	131.0 (7)
C4—C3—C2	107.0 (5)	C4A—C5A—H5D	109.5
C4—C3—H3A	126.5	C4A—C5A—H5E	109.5
C2—C3—H3A	126.5	H5D—C5A—H5E	109.5
N2—C4—C3	107.0 (5)	C4A—C5A—H5F	109.5
N2—C4—C5	122.5 (5)	H5D—C5A—H5F	109.5
C3—C4—C5	130.5 (5)	H5E—C5A—H5F	109.5
N2—C6—C7	110.8 (4)	N2A—C6A—C7A	109.7 (5)
N2—C6—H6A	109.5	N2A—C6A—H6C	109.7
C7—C6—H6A	109.5	C7A—C6A—H6C	109.7
N2—C6—H6B	109.5	N2A—C6A—H6D	109.7
C7—C6—H6B	109.5	C7A—C6A—H6D	109.7
H6A—C6—H6B	108.1	H6C—C6A—H6D	108.2
N3—C7—C6	111.2 (4)	C6A—C7A—N3A	111.5 (6)
N3—C7—H7A	109.4	C6A—C7A—H7C	109.3
C6—C7—H7A	109.4	N3A—C7A—H7C	109.3
N3—C7—H7B	109.4	C6A—C7A—H7D	109.3
C6—C7—H7B	109.4	N3A—C7A—H7D	109.3
H7A—C7—H7B	108.0	H7C—C7A—H7D	108.0
N3—C8—C9	111.6 (6)	N3A—C8A—C9A	111.3 (8)
N3—C8—H8A	109.3	N3A—C8A—H8C	109.4
C9—C8—H8A	109.3	C9A—C8A—H8C	109.4
N3—C8—H8B	109.3	N3A—C8A—H8D	109.4
C9—C8—H8B	109.3	C9A—C8A—H8D	109.4
H8A—C8—H8B	108.0	H8C—C8A—H8D	108.0
N4—C9—C8	111.4 (7)	N4A—C9A—C8A	112.0 (10)
N4—C9—H9A	109.3	N4A—C9A—H9C	109.2
C8—C9—H9A	109.3	C8A—C9A—H9C	109.2
N4—C9—H9B	109.3	N4A—C9A—H9D	109.2
C8—C9—H9B	109.3	C8A—C9A—H9D	109.2
H9A—C9—H9B	108.0	H9C—C9A—H9D	107.9
N4—C11—C12	105.8 (5)	C11A—C10A—H10D	109.5
N4—C11—C10	123.8 (5)	C11A—C10A—H10E	109.5
C12—C11—C10	130.4 (4)	H10D—C10A—H10E	109.5
C11—C12—C13	107.0 (5)	C11A—C10A—H10F	109.5
C11—C12—H12	126.5	H10D—C10A—H10F	109.5
C13—C12—H12	126.5	H10E—C10A—H10F	109.5
N5—C13—C12	109.7 (6)	N4A—C11A—C12A	107.7 (7)
N5—C13—C14	121.7 (6)	N4A—C11A—C10A	122.5 (6)
C12—C13—C14	128.2 (6)	C12A—C11A—C10A	129.8 (7)
N1A—Pd1A—N5A	170.5 (6)	C11A—C12A—C13A	108.2 (7)
N1A—Pd1A—N3A	90.8 (4)	C11A—C12A—H12A	125.9
N5A—Pd1A—N3A	84.5 (7)	C13A—C12A—H12A	125.9
N1A—Pd1A—Cl1A	91.72 (13)	C12A—C13A—N5A	107.1 (8)
N5A—Pd1A—Cl1A	93.9 (6)	C12A—C13A—C14A	130.8 (7)
N3A—Pd1A—Cl1A	173.5 (4)	N5A—C13A—C14A	122.1 (8)
C2A—N1A—N2A	107.5 (5)	C13A—C14A—H14D	109.5
C2A—N1A—Pd1A	133.8 (4)	C13A—C14A—H14E	109.5
N2A—N1A—Pd1A	118.5 (4)	H14D—C14A—H14E	109.5
C4A—N2A—N1A	109.8 (6)	C13A—C14A—H14F	109.5
C4A—N2A—C6A	128.9 (6)	H14D—C14A—H14F	109.5
N1A—N2A—C6A	119.3 (6)	H14E—C14A—H14F	109.5
			
N3—Pd1—N1—C2	−148.4 (4)	N3A—Pd1A—N1A—C2A	−138.0 (6)
Cl1—Pd1—N1—C2	34.7 (4)	Cl1A—Pd1A—N1A—C2A	48.0 (4)
N3—Pd1—N1—N2	49.4 (3)	N3A—Pd1A—N1A—N2A	47.0 (5)
Cl1—Pd1—N1—N2	−127.5 (2)	Cl1A—Pd1A—N1A—N2A	−127.0 (3)
C2—N1—N2—C4	0.6 (4)	C2A—N1A—N2A—C4A	0.0 (5)
Pd1—N1—N2—C4	167.4 (2)	Pd1A—N1A—N2A—C4A	176.2 (3)
C2—N1—N2—C6	167.5 (3)	C2A—N1A—N2A—C6A	165.0 (4)
Pd1—N1—N2—C6	−25.7 (4)	Pd1A—N1A—N2A—C6A	−18.7 (5)
N1—Pd1—N3—C8	120.0 (8)	N1A—Pd1A—N3A—C7A	−18.1 (9)
N5—Pd1—N3—C8	−64.6 (9)	N5A—Pd1A—N3A—C7A	170.2 (11)
N1—Pd1—N3—C7	−15.0 (7)	N1A—Pd1A—N3A—C8A	110.8 (9)
N5—Pd1—N3—C7	160.4 (8)	N5A—Pd1A—N3A—C8A	−60.9 (11)
C11—N4—N5—C13	3.8 (17)	C11A—N4A—N5A—C13A	−7(2)
C9—N4—N5—C13	−176.5 (11)	C9A—N4A—N5A—C13A	176.1 (14)
C11—N4—N5—Pd1	−175.1 (8)	C11A—N4A—N5A—Pd1A	−165.3 (12)
C9—N4—N5—Pd1	4.6 (18)	C9A—N4A—N5A—Pd1A	18 (2)
N3—Pd1—N5—C13	−129.7 (19)	N3A—Pd1A—N5A—N4A	39.0 (17)
Cl1—Pd1—N5—C13	47.5 (18)	Cl1A—Pd1A—N5A—N4A	−147.3 (17)
N3—Pd1—N5—N4	48.7 (12)	N3A—Pd1A—N5A—C13A	−113 (2)
Cl1—Pd1—N5—N4	−134.1 (12)	Cl1A—Pd1A—N5A—C13A	61 (2)
N2—N1—C2—C3	0.2 (4)	N2A—N1A—C2A—C3A	0.4 (5)
Pd1—N1—C2—C3	−163.5 (3)	Pd1A—N1A—C2A—C3A	−175.0 (4)
N2—N1—C2—C1	−174.3 (3)	N2A—N1A—C2A—C1A	−177.0 (4)
Pd1—N1—C2—C1	22.0 (5)	Pd1A—N1A—C2A—C1A	7.6 (7)
N1—C2—C3—C4	−0.9 (4)	N1A—C2A—C3A—C4A	−0.6 (6)
C1—C2—C3—C4	173.2 (4)	C1A—C2A—C3A—C4A	176.5 (5)
N1—N2—C4—C3	−1.2 (4)	N1A—N2A—C4A—C3A	−0.4 (6)
C6—N2—C4—C3	−166.4 (4)	C6A—N2A—C4A—C3A	−163.6 (5)
N1—N2—C4—C5	178.4 (4)	N1A—N2A—C4A—C5A	179.9 (5)
C6—N2—C4—C5	13.1 (6)	C6A—N2A—C4A—C5A	16.7 (9)
C2—C3—C4—N2	1.2 (4)	C2A—C3A—C4A—N2A	0.6 (6)
C2—C3—C4—C5	−178.2 (4)	C2A—C3A—C4A—C5A	−179.7 (6)
C4—N2—C6—C7	121.7 (5)	C4A—N2A—C6A—C7A	112.8 (7)
N1—N2—C6—C7	−42.5 (5)	N1A—N2A—C6A—C7A	−49.0 (7)
C8—N3—C7—C6	−174.3 (6)	N2A—C6A—C7A—N3A	78.9 (9)
Pd1—N3—C7—C6	−39.0 (8)	C8A—N3A—C7A—C6A	−166.3 (7)
N2—C6—C7—N3	79.1 (7)	Pd1A—N3A—C7A—C6A	−34.8 (11)
C7—N3—C8—C9	160.3 (6)	C7A—N3A—C8A—C9A	160.0 (7)
Pd1—N3—C8—C9	24.4 (9)	Pd1A—N3A—C8A—C9A	27.7 (12)
C11—N4—C9—C8	112.6 (12)	N5A—N4A—C9A—C8A	−73.7 (19)
N5—N4—C9—C8	−67.0 (14)	C11A—N4A—C9A—C8A	109.9 (16)
N3—C8—C9—N4	46.6 (9)	N3A—C8A—C9A—N4A	44.1 (12)
N5—N4—C11—C12	−2.5 (13)	N5A—N4A—C11A—C12A	2.8 (18)
C9—N4—C11—C12	177.9 (11)	C9A—N4A—C11A—C12A	179.4 (15)
N5—N4—C11—C10	175.0 (9)	N5A—N4A—C11A—C10A	−178.8 (13)
C9—N4—C11—C10	−4.6 (16)	C9A—N4A—C11A—C10A	−2(2)
N4—C11—C12—C13	0.3 (8)	N4A—C11A—C12A—C13A	2.6 (11)
C10—C11—C12—C13	−177.0 (5)	C10A—C11A—C12A—C13A	−175.7 (8)
N4—N5—C13—C12	−3.6 (15)	C11A—C12A—C13A—N5A	−6.7 (14)
Pd1—N5—C13—C12	174.9 (14)	C11A—C12A—C13A—C14A	175.5 (9)
N4—N5—C13—C14	−176.6 (9)	N4A—N5A—C13A—C12A	8(2)
Pd1—N5—C13—C14	2(2)	Pd1A—N5A—C13A—C12A	162.4 (15)
C11—C12—C13—N5	2.2 (11)	N4A—N5A—C13A—C14A	−173.8 (13)
C11—C12—C13—C14	174.6 (6)	Pd1A—N5A—C13A—C14A	−20 (3)

### Hydrogen-bond geometry (Å, °)

**Table:**

*D*—H···*A*	*D*—H	H···*A*	*D*···*A*	*D*—H···*A*
N3—H3···Cl2	0.93	2.24	3.162 (12)	174
N3A—H3B···Cl2A	0.93	2.17	3.088 (15)	170
